# Glycoproteomic profiling of serum-derived small extracellular vesicles enriched via ultracentrifugation and affinity-based techniques

**DOI:** 10.1038/s41598-025-05430-1

**Published:** 2025-07-01

**Authors:** Mojibola Fowowe, Cristian D. Gutierrez Reyes, Judith Nwaiwu, Joy Solomon, Oluwatosin Daramola, Sherifdeen Onigbinde, Joseph Andrew Whitley, Houjian Cai, Yehia Mechref

**Affiliations:** 1 Department of Chemistry and Biochemistry, Texas Tech University, Lubbock, TX 79409-1061 USA; 2 Department of Pharmaceutical and Biomedical Sciences, College of Pharmacy, University of Georgia, Athens, GA 30602 USA

**Keywords:** Small extracellular vesicles, EVs, Immunoaffinity capture, Ultracentrifugation, Glycoproteomics, Proteomics, Biotechnology, Proteomics

## Abstract

Small extracellular vesicles (sEVs) are gaining recognition as potential biomarkers for diseases, including cancer, due to their involvement in key pathophysiological processes. However, the glycosylation of EVs and the specific roles of their glycans remain poorly understood. While several methods exist for isolating sEVs from complex biological samples, achieving sufficient purity and quantity for mass spectrometry-based glycoproteomic analysis remains a significant challenge. In this study, we compared two commonly used isolation methods, ultracentrifugation (UC) and immunoaffinity capture (MagCapture kit), across different starting volumes of human serum (200 µL and 500 µL) to evaluate their performance for downstream glycoproteomic analysis. While prior studies have examined protein content across isolation methods, our work uniquely investigates how isolation technique and sample volume affect glycoproteomic yield and quality. We show that UC, particularly at higher sample volumes, enables deeper glycoproteomic coverage, whereas MagCapture is advantageous when serum availability is limited. Notably, we report for the first time site-specific glycan microheterogeneity on sEV glycoproteins derived from human serum, including multiple glycoforms at the same glycosylation site. These findings highlight the complexity and biological relevance of glycosylation in sEV proteins and offer practical guidance for optimizing isolation protocols based on specific omics applications.

**Supplementary Information:**

The online version contains supplementary material available at 10.1038/s41598-025-05430-1.

## Introduction

Small extracellular vesicles (sEVs) are a subpopulation of extracellular vesicles that promise potential for defining reliable biomarkers and developing targeted therapeutics^1–3^. These vesicles, ranging in size from 40 to 200 nm, are secreted by most cell types and have been identified in nearly all bodily fluids, including saliva, urine, blood, breast milk, and cerebrospinal fluid^4–7^. EVs were first described in the 1980s by Stahl and Johnstone as vesicles that remove unwanted proteins from the cell cytoplasm^8^. However, over time, their more complex roles have come to light. EVs carry many vital biomolecules, such as proteins, glycans, nucleic acids, lipids, and metabolites from their parent cells^9,10^, and they facilitate the transfer of these contents to neighboring cells This intercellular communication is crucial in numerous physiological and pathological processes, including embryonic development, immune responses, tissue regeneration, vascular homeostasis, and the progression of diseases such as cancer^11–18^.

A key subset of sEVs, exosomes, have been particularly well studied. Their biogenesis begins with the inward budding of the plasma membrane, forming early endosomes. These endosomes invaginate and generate intraluminal vesicles (ILVs) within multivesicular bodies (MVBs). When MVBs fuse with the plasma membrane, exosomes are released into the extracellular space^19–24^.

Despite the growing interest in sEVs, research has been hindered by the need for standardized isolation protocols. A method that reliably isolates pure sEV populations with high yield is essential for accurate profiling. Even with recent progress, much of the existing data comes from heterogeneous or contaminated sEVs preparations, complicating findings^1,25^. While ultracentrifugation (UC) is widely used, it is time-consuming and may co-isolate protein aggregates or non-EV particles, compromising purity and reproducibility^26^.

To address these issues, alternative isolation methods have been developed, including commercial kits that apply principles such as charge, size, shape, and immunoaffinity with membrane surface markers like CD9, CD63, and CD81. A common method involves polymer-based reagents like polyethylene glycol (PEG), which can provide a high yield of sEVs but may also introduce contaminants that interfere with downstream mass spectrometry applications^27–32^. However, harsh elution conditions required by these methods may compromise the integrity of the isolated vesicles, limiting their utility for structural and functional studies^33^. In response to this challenge, Nakai et al.^34^ developed a method using T-cell immunoglobulin domain and mucin domain-containing protein 4 (Tim4) to bind phosphatidylserine (PS) on the sEV surface, enabling the isolation of purer, intact vesicles with fewer contaminants than conventional methods.

Numerous studies have compared different sEV isolation techniques, primarily focusing on vesicular RNA, proteins, and intact sEVs^35–39^. Collectively, these studies have shown that vesicle yield, purity, and molecular content can vary considerably depending on the isolation method, presenting challenges for the reproducibility and standardization of EV research. In contrast, investigations into the glycoproteomic profiles of sEVs remain limited, despite growing recognition of the roles EV glycans play in pathophysiological processes^40–43^. Comparative glycoproteomic studies, particularly those involving serum-derived sEVs isolated using different methods, are needed to understand how isolation protocols impact glycoprotein content and to advance the development of reliable biomarker discovery workflows.

Glycosylation, a common post-translational modification (PTM) of proteins, regulates several biological processes such as cell signaling, cell adhesion, immune cell trafficking, and protein stability^44–52^. The two most prevalent forms of glycosylation are *N*-glycosylation, which occurs on asparagine residues within the Asp-X-Ser/Thr motif, and *O*-glycosylation, which occurs on serine or threonine residues without a defined target motif. Recent research has highlighted the role of EV glycosylation in identifying cancer-related glycomarkers, suggesting a novel avenue for cancer diagnosis^53,54^.

In this study, we compared the glycoproteome of serum-derived sEVs isolated using two different methods: ultracentrifugation (UC) and immunoaffinity capture with the MagCapture Exosome Isolation Kit Ver. 2. We assessed the impact of sample volume on the efficiency of these methods by testing serum samples of 200 µL and 500 µL. We aimed to determine the optimal and most adaptable workflow for serum sEV glycoproteomic studies, providing a reliable and robust method for future research.

## Materials and methods

### Chemicals and reagents

Sterile-filtered human serum (HS) from human male AB plasma, iodoacetamide (IAA), dithiothreitol (DTT), ammonium bicarbonate (ABC), trifluoroacetic acid (TFA), and sodium deoxycholate (SDC) were obtained from Sigma-Aldrich (St. Louis, MO, USA). MagCapture Exosome Isolation Kit PS Ver. 2 (Mag) was purchased from Fujifilm Wako Chemicals U.S.A Corporation (Richmond, VA, USA). Mass spectrometry grade trypsin/lys-C mix was sourced from Promega (Madison, WI, USA). HPLC-grade submicron filtered water, acetonitrile (MeCN), and formic acid (FA) were acquired from Fisher Scientific (Fair Lawn, NJ, USA). The protein micro-BCA assay kit was procured from Thermo Scientific (Rockford, IL, USA). PolyHYDROXYETHYL A HILIC spin columns for glycopeptide enrichment were obtained from Poly LC INC. (Columbia, MD, USA). Particle-free phosphate-buffered saline (PBS) 1X, was purchased from Mediatech Inc. (Manassas, VA, USA).

### Enrichment of sEVs

#### Rationale for sample volume selection

The selection of 200 µL and 500 µL serum volumes for sEV isolation was guided by both practical and methodological considerations. These volumes reflect sample quantities commonly available in clinical and translational research, particularly when working with biobanked specimens or limited patient-derived samples. To evaluate the feasibility of low-input workflows, preliminary assessments were conducted using smaller volumes (50 µL and 100 µL) with the MagCapture kit. However, these volumes did not yield sufficient vesicular material for downstream proteomic and glycoproteomic analyses, limiting their utility for comprehensive profiling. Accordingly, 200 µL was selected to evaluate method performance under low-volume conditions, while 500 µL served as a higher-input benchmark for assessing potential gains in sEV yield and glycoproteome coverage. For UC, the input volume was limited to 200 µL due to known sample volume constraints and supporting precedent in the literature^35,55^. The comparative findings across these volumes provided insight into the balance between input quantity and analytical depth, informing future protocol optimization.

#### Sample pre-treatment

Human serum samples (200 µL and 500 µL) were initially centrifuged at 300×*g* for 10 min at 4 °C to remove cells. The resulting supernatant was then centrifuged at 2000×*g* for 10 min at 4 °C to eliminate cellular debris, followed by a third centrifugation at 10,000×*g* for 30 min at 4 °C to eliminate larger EVs. The final supernatant was filtered through a sterile 0.22 µm filter to further exclude residual large EVs and particles. Small extracellular vesicles (sEVs) were subsequently isolated from the 10 K filtrates using either UC or the MagCapture affinity-based method. All experiments were performed in triplicate.

#### Ultracentrifugation isolation

The 10 K filtrate from the previous step was diluted with ice-cold 1X PBS to a final volume of 2 mL, transferred into Ultra-Clear centrifuge tubes, and centrifuged at 100,000×*g* for 70 min at 4 °C using a Beckman Optima XE-90 ultracentrifuge with a Type 70.1 Ti fixed-angle rotor (Beckman Coulter, Indianapolis, IN). After centrifugation, the supernatant was carefully removed with a pipette, and the pellet was washed with ice-cold 1X PBS. The sample was centrifuged at 100,000×*g* for 70 min at 4 °C. Finally, the pelleted sEVs were resuspended in 200 µL of 1X PBS and stored at − 80 °C for future use. All ultracentrifugation steps were performed with maximum acceleration and without braking during deceleration.

#### Affinity-based isolation

sEVs were isolated from the 10 K filtrate using the MagCapture Exosome Isolation Kit PS Ver. 2 (Mag), following the manufacturer’s protocol. Briefly, the 10 K filtrate was diluted to 700 µL with Tris-buffered saline (TBS) and incubated with magnetic beads prepped for sEV capture through an affinity reaction. The mixture was gently agitated overnight at 4 °C on an Eppendorf ThermoMixer. Following incubation, the sEV-bound beads were washed three times with the provided wash buffer before elution. sEVs were eluted twice from the beads using 50 µL of the elution buffer, and the eluates were combined. The pooled eluates were then stored at − 80 °C for future analysis.

### sEVs characterization by transmission electron microscopy (TEM)

The sEV sample was drop-mounted onto carbon-coated grids for 1 min. Excess sample was carefully blotted off with lint-free filter paper, followed by negative staining with freshly prepared 1% uranyl acetate for 30 s. The excess stain was then blotted off, allowing the grid to air dry completely. Once dried, the grids were imaged using a Hitachi H-7650 transmission electron microscope (Hitachi High-Tech America, Inc., USA) operating at an accelerating voltage of 100 kV.

### sEVs characterization by nano flow cytometry

The concentration and size distribution of sEVs were determined using nanoparticle flow cytometry with the NanoAnalyzer instrument (NanoFCM). The system was calibrated for particle concentration using 250 nm SiNP QC beads and for size distribution using the S16M-Exo silica nanosphere cocktail, diluted 1:100. The sEVs were unlabeled and diluted with PBS as needed to achieve a concentration of 1 × 10^8^ to 1 × 10^9^ particles per mL for accurate measurement. Data on size distribution and particle concentration were analyzed using NanoFCM Software V1.17.

### sEVs lysis and protein content determination

The sEVs were lysed using a 5% SDC detergent solution and mechanically disrupted with a Beadbug microtube homogenizer (Benchmark Scientific, Edison, NJ, USA), following a previously described protocol^56,57^. Briefly, approximately 100 mg of 400 µm zirconium beads (molecular biology grade) and 100 µL of 5% SDC were combined with 100 µL of the extracted sEV sample in a 2 mL microcentrifuge tube. The homogenization was performed using the Beadbug device, set to agitate at 4000 rpm for 5 cycles (30 s per cycle) with 30-s intervals between cycles to prevent overheating.

After homogenization, the samples were sonicated for 1 h in a bath sonicator, with the tubes placed in an ice-water slurry to maintain low temperature and prevent heat-induced degradation. Following sonication, the samples were centrifuged at 14,800 rpm for 10 min at 4 °C to collect the supernatant. Formic acid (FA) was then added to the supernatants to a final concentration of 0.5% to precipitate the SDC detergent. The resulting acidic mixture was vortexed thoroughly and centrifuged at 1000 rpm for 1 min, followed by a second centrifugation at 14,800 rpm for 10 min. The clear supernatant was carefully collected and dried using a SpeedVac vacuum concentrator. The dried samples were resuspended in 50 µL of 50 mM ABC buffer and aliquoted for protein quantification using the Micro-BCA Protein Assay Kit (Thermo Scientific/Pierce, Rockford, IL), according to the manufacturer’s instructions.

### Tryptic digestion of sEVs proteins

The protein samples were first thermally denatured at 90 °C for 15 min, followed by reduction with 200 mM DTT at 60 °C for 45 min. After reduction, proteins were alkylated with 200 mM IAA at 37 °C for 45 min in the dark. To quench residual IAA, a second aliquot of 200 mM DTT was added, and the samples were incubated at 37 °C for an additional 30 min. Next, a trypsin/Lys-C protease mix was added at a 25:1 protein-to-enzyme ratio and incubated overnight at 37 °C. The inclusion of Lys-C helps reduce the miscleavages commonly associated with trypsin digestion. Following digestion, the samples were divided into two aliquots and vacuum-dried. One aliquot was resuspended in an aqueous solution containing 2% acetonitrile (MeCN) and 0.1% formic acid (FA) for LC–MS/MS proteomic analysis. The other aliquot was utilized for *N*-glycopeptide enrichment as described in the next protocol.

### Off-line HILIC enrichment of *N*-glycopeptides

Due to the low protein content recovered from serum-derived sEVs, peptide desalting step was omitted to minimize sample loss and maximize glycopeptide recovery. This streamlined approach has been validated in prior glycoproteomics studies by our group, where efficient enrichment and high-quality data were achieved without desalting^58–60^.

*N*-glycopeptide enrichment was performed using HILIC TopTip PolyHYDROXYETHYL A spin columns (PolyLC Inc., Columbia, MD) following a previously described protocol^61^. Briefly, vacuum-dried peptides were resuspended in 50 µL of a loading buffer containing 80% acetonitrile (MeCN) and 1% trifluoroacetic acid (TFA). The HILIC TopTip columns were conditioned by washing three times with 100 µL of water, followed by 100 µL of the loading buffer at 1000×*g* for 1 min.

The resuspended peptide samples were then loaded onto the conditioned columns and centrifuged at 500×*g* for 20 s. After a 5-min incubation, the flow-through was collected, and the sample was reloaded onto the column three more times to ensure complete binding of the glycopeptides. The columns were then washed three times with 100 µL of loading buffer.

Finally, the glycopeptides were eluted from the columns three times using 100 µL of 1% TFA, spun at 1000×*g* for 1 min. The enriched glycopeptides were vacuum-dried and resuspended in an aqueous solution of 2% MeCN and 0.1% FA for subsequent LC–MS/MS glycoproteomic analysis.

### Quantitative LC–MS/MS proteomic and *N*-glycoproteomic analysis

LC–MS/MS analysis was performed using a Dionex Ultimate 3000 nano-LC system coupled to a Q Exactive HF—Orbitrap mass spectrometer (Thermo Scientific, San Jose, CA), equipped with a nano-electrospray ionization (nano-ESI) source. Each sample injection contained 1 µg of protein, as the micro-BCA protein assay determined. Peptides were first desalted online using a reverse-phase C18 Acclaim PepMap trap column (75 μm × 2 mm, 3 μm, 100 Å, Thermo Scientific) before being separated on a C18 Acclaim PepMap RSLC analytical column (75 μm i.d. × 15 cm, 2 μm, 100 Å, Thermo Scientific). The flow rate was maintained at 350 nL/min, with the column temperature at 29.5 °C.

Mobile phase A (MPA) consisted of 2% acetonitrile (MeCN) with 0.1% formic acid (FA), while mobile phase B (MPB) was 0.1% FA in MeCN. The chromatographic gradient started with 2% MPB for the first 10 min, then a linear increase to 20% over the next 55 min. MPB was then ramped from 30 to 50% between 90 and 110 min. The gradient was held at 80% MPB for 5 min, then rapidly reduced to 2% at 116 min, which was maintained to equilibrate the column.

The Q Exactive HF—Orbitrap mass spectrometer operated in positive ion mode with an electrospray ionization voltage of 1.6 kV. Two scan events were performed: a data-dependent full MS scan within the 400–2000 m/z range at a mass resolution of 120,000, with an AGC target of 1 × 10^6^ and a maximum injection time of 100 ms. This was followed by a data-dependent MS^2^ (ddMS^2^) scan, in which the top 20 most intense ions from the full MS scan were selected for high-energy collision-induced dissociation (HCD) fragmentation. A stepped normalized collision energy (CE) of 15, 25, and 35 was applied for fragmentation. Fragment ions within the mass range of 200–2000 m/z were detected at a resolution of 45,000, with an AGC target of 1 × 10^5^ and a maximum injection time of 50 ms.

### Data analysis

Raw data from the LC–MS/MS runs were processed using the SEQUEST search engine in Proteome Discoverer (PD) Software v2.5 (Thermo Scientific, San Jose, CA) for proteomic analysis and Byonic Software (Protein Metrics, Cupertino, CA) for *N*-glycoproteomic analysis. Two database searches were performed.

The first search utilized a curated target list of the top 100 EV proteins from the ExoCarta database^62^ to reduce the search space, minimizing random hits and providing more confident identification of precursor and fragment ions using the high-resolution Orbitrap mass analyzer. The second search was conducted against the UniProt KB/Swiss-Prot human protein database (TaxId:9609).

For both search engines, carbamidomethylation of cysteine was set as a fixed modification, while oxidation of methionine and acetylation at the protein *N*-terminus were set as variable modifications. Peptide and glycopeptide precursor mass tolerances were set to 10 ppm, with fragment mass tolerances of 0.02 Da for PD and 20 ppm for Byonic. Trypsin was specified as the proteolytic enzyme, allowing for up to two missed cleavages.

In PD, peptide-spectral matches (PSMs) were validated using Percolator with a 1% false discovery rate (FDR) cut-off^63,64^. Identified peptides were grouped into proteins according to the law of parsimony and filtered based on a 1% FDR threshold. For Byonic, QTOF/HCD fragmentation was selected, and a default list of 132 mammalian glycans was used for glycopeptide identification. The results were also filtered at a 1% FDR threshold. Additionally, identified sEV proteins were annotated with Gene Ontology (GO) terms using FunRich software v3.1.3.

Quantitative glycopeptide abundance for relative comparisons was derived from peak area values extracted using Thermo XCalibur software, followed by log₂ transformation to facilitate comparison across different sEV isolation methods.

All analyses were conducted in triplicate. To minimize handling variability, all samples were processed in parallel following the same protocol. Variation across replicates was assessed by evaluating standard deviations in particle size, protein yield, and identified glycoproteins/glycopeptides. Statistical analysis was performed using a two-tailed unpaired Student’s t-test, with a significance threshold set at *p* < 0.05.

## Results

### Experimental workflow for sEV isolation and characterization

**Fig. 1:**
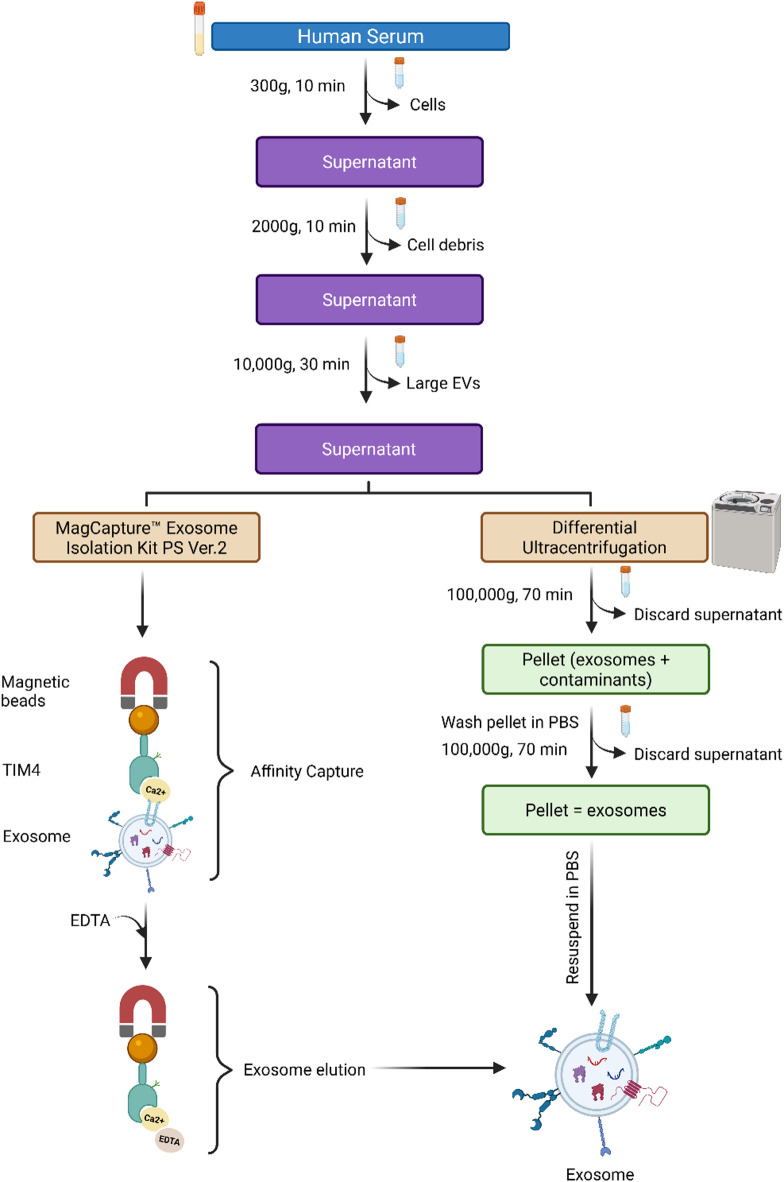
Workflow for sEV isolation from human serum using the MagCapture Exosome Isolation Kit PS Ver. 2 (Mag) and differential ultracentrifugation (UC). Serum samples were first subjected to sequential centrifugation steps to remove cells, cellular debris, and larger vesicles, followed by either affinity-based capture (Mag) or ultracentrifugation (UC) for sEV enrichment.

The workflow for sEV isolation and characterization used in this study is illustrated in Fig. 1. sEVs were extracted from two different starting volumes (200 µL and 500 µL) of standard human serum using two distinct methods: differential ultracentrifugation (UC) and the MagCapture Exosome Isolation Kit PS Ver. 2 (Mag). Following the MISEV2018 guidelines and the recommendations by Théry et al. for EV characterization, the isolated sEVs were comprehensively characterized^65^.

**Fig. 2:**
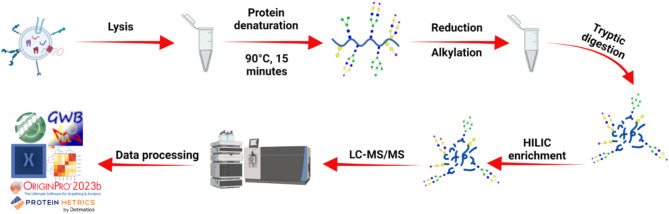
Workflow for glycoproteomic sample preparation. Isolated sEVs were lysed using a 5% SDC detergent solution with bead beating and ultrasonication. The released proteins were then denatured, reduced, alkylated, and digested with trypsin. Glycosylated peptides were subsequently enriched using HILIC and analyzed by LC–MS/MS.

Transmission electron microscopy (TEM) was employed to evaluate the size and morphology of the vesicles, while nano-flow cytometry (nFCM) was used to verify particle concentration and size distribution. Proteomic characterization was achieved through unbiased, high-sensitivity LC–MS/MS analysis of the sEV cargo. Subsequently, *N*-glycoproteomic profiling was performed, as shown in Fig. 2, to explore the glycoproteome of the vesicles. The results were then compared to identify similarities and differences between the *N*-glycoproteomes of sEVs isolated by different methods. For clarity, each experiment was designated by its isolation method and starting sample volume. For instance, the ultracentrifugation method using a 200 µL sample is referred to as UC-200.

#### High-resolution TEM confirms size and morphology of sEVs isolated by different methods

TEM was used to visualize and confirm the characteristic size and morphology of sEVs isolated by the two methods. TEM analysis of sEVs from each experiment confirmed the presence of vesicles with the typical cup-shaped or round morphology, consistent with the expected membrane lipid bilayer structure^66–68^.

**Fig. 3:**
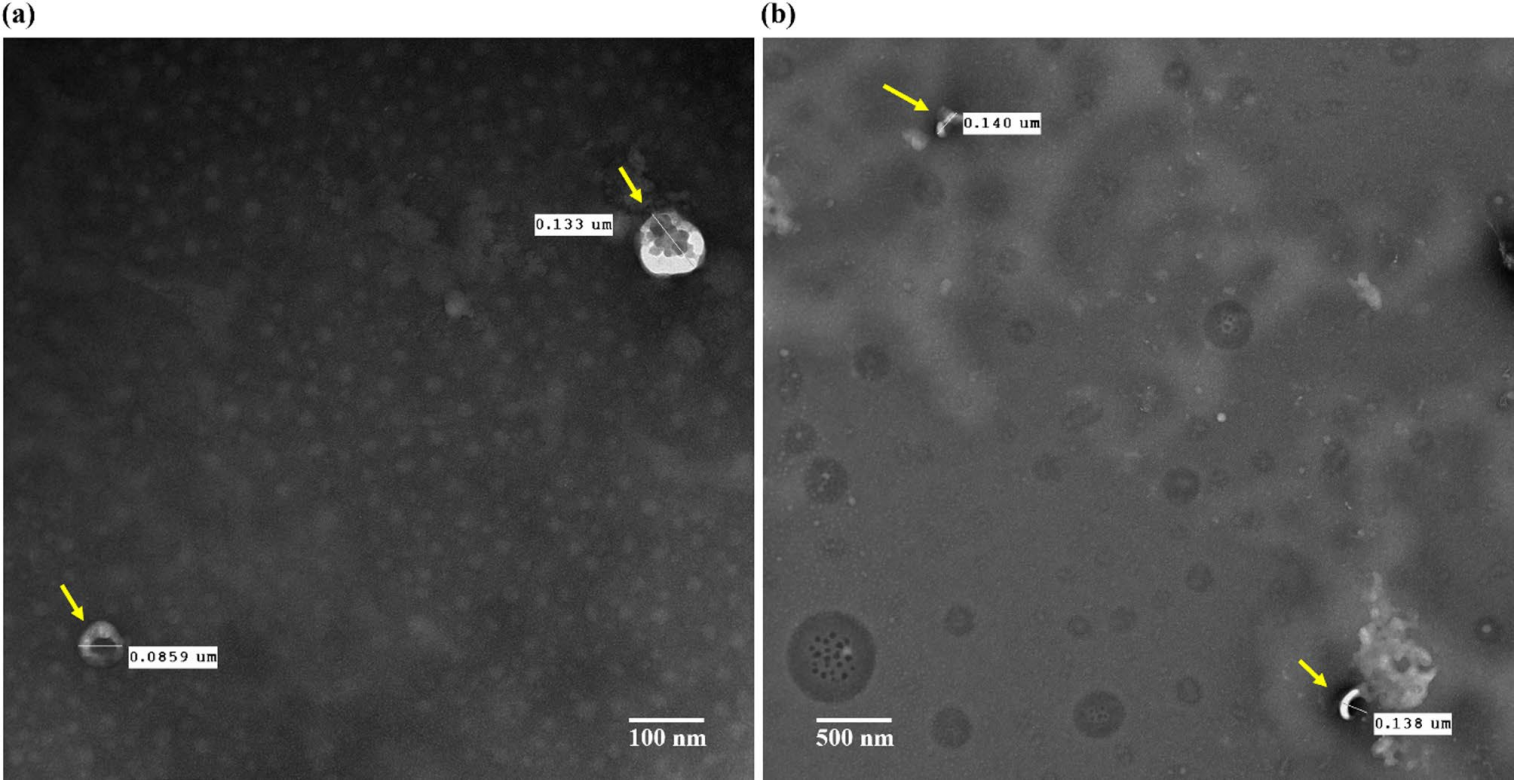
Representative transmission electron micrograph of sEVs isolated from human serum using the (**a**) the MagCapture affinity method (Mag-200) and (**b**) the ultracentrifugation method (UC-500). TEM analysis confirmed the characteristics round or cup-shaped morphology of sEVs and verified that their sizes fell within the typical range of 40–200 nm.

Both UC and Mag methods yielded sEVs within the standard size range of 40–200 nm, as shown in the TEM images for Mag (Fig. 3a) and UC (Fig. 3b). Notably, the UC-500 samples displayed a broader size distribution compared to their Mag counterparts, suggesting that ultracentrifugation may enrich a more heterogeneous population of vesicles (Fig. 3b).

These results demonstrate that both isolation methods effectively recover sEVs with typical morphologies, though differences in size distributions may reflect variations in the populations isolated by each technique.

#### Impact of isolation technique and sample volume on sEV particle concentration and size distribution

**Fig. 4:**
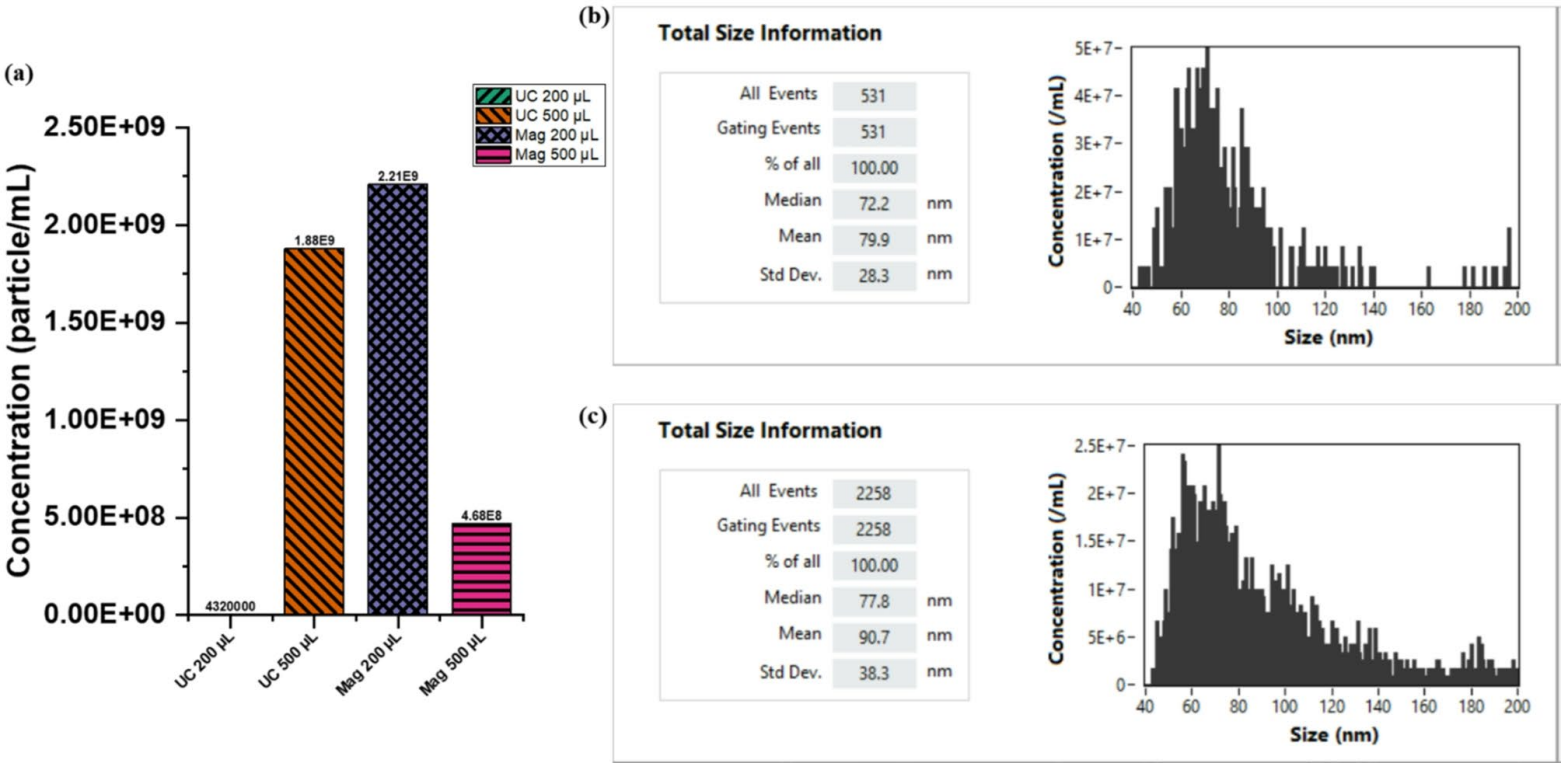
(**a**) Concentration of nanovesicles within the typical 40 – 200 nm size range of sEVs in the isolated fractions, and representative nFCM particle size distribution (median and mean) results of (**b**) Mag-200 and (**c**) UC-500 sEVs fractions. Due to the low starting sample volumes, particle concentrations were too low to allow reliable quantification across biological replicates. Therefore, all data shown represent a single measurement per condition. Particle size values reflect intra-sample variability based on gated events. Among the methods, Mag-200 produced the highest particle count, followed by UC-500, Mag-500, and UC-200. UC-500 particles exhibited a larger size distribution compared to those from Mag-200.

The concentration and size distribution of sEVs can be influenced by both the isolation method and the starting sample volume. To evaluate these effects in serum-derived sEVs, nano-flow cytometry (nFCM) was employed to estimate particle concentrations and measure median and mean size distributions. The results are summarized in Table 1 and Fig. 4, with detailed reports provided in Online Resource 1–4.

**Table 1:** Concentration and size distribution of nanovesicles produced by different isolation methods, as determined by nano-flow cytometry (nFCM).

	Particles/mL	Median (nm)	Mean (nm)	Std Dev. (nm)
UC-200	4.32E+06	52.2	71.6	48.3
UC-500	1.88E+09	77.8	90.7	38.3
Mag-200	2.21E+09	72.2	79.9	28.3
Mag-500	4.68E+08	77.2	90.6	37.8

Particle concentration, expressed as particles/mL, varied across the different methods. Mag-200 yielded the highest concentration, followed by UC-500, Mag-500, and UC-200 (Fig. 4a). All isolation methods produced particles within the expected size range of 40–200 nm, although median and mean sizes varied.

Specifically, Mag-200 produced a median particle size of 72.2 nm and a mean size of 79.9 nm (Fig. 4b). Mag-500 yielded a median size of 77.2 nm and a mean of 90.6 nm. UC-200 generated smaller vesicles, with a median size of 52.2 nm and a mean size of 71.6 nm, while UC-500 produced larger vesicles, with a median size of 77.8 nm and a mean of 90.7 nm (Fig. 4c).

Overall, UC-500 and Mag-500 yielded the largest median and mean particle sizes, whereas UC-200 produced the smallest particles. These findings emphasize the influence of both the isolation technique and sample volume on the physical characteristics of serum-derived sEVs.

Due to the low starting sample volumes, particle concentrations were too low to yield reliable measurements across biological replicates. As a result, both particle size values (mean and median) and concentration values are reported from a single measurement and reflect intra-sample variability based on gated events.

#### Protein yield and evaluation of sEV purity using proteomic and glycoproteomic markers

Downstream LC–MS/MS analysis of sEVs is often constrained by the low protein yield from enriched fractions. However, higher protein content does not always correlate with purer sEV fractions, as contaminants such as high-abundance proteins can co-isolate with sEVs, complicating the interpretation of results. Moreover, the type and level of contamination can vary depending on the isolation method.

**Fig. 5:**
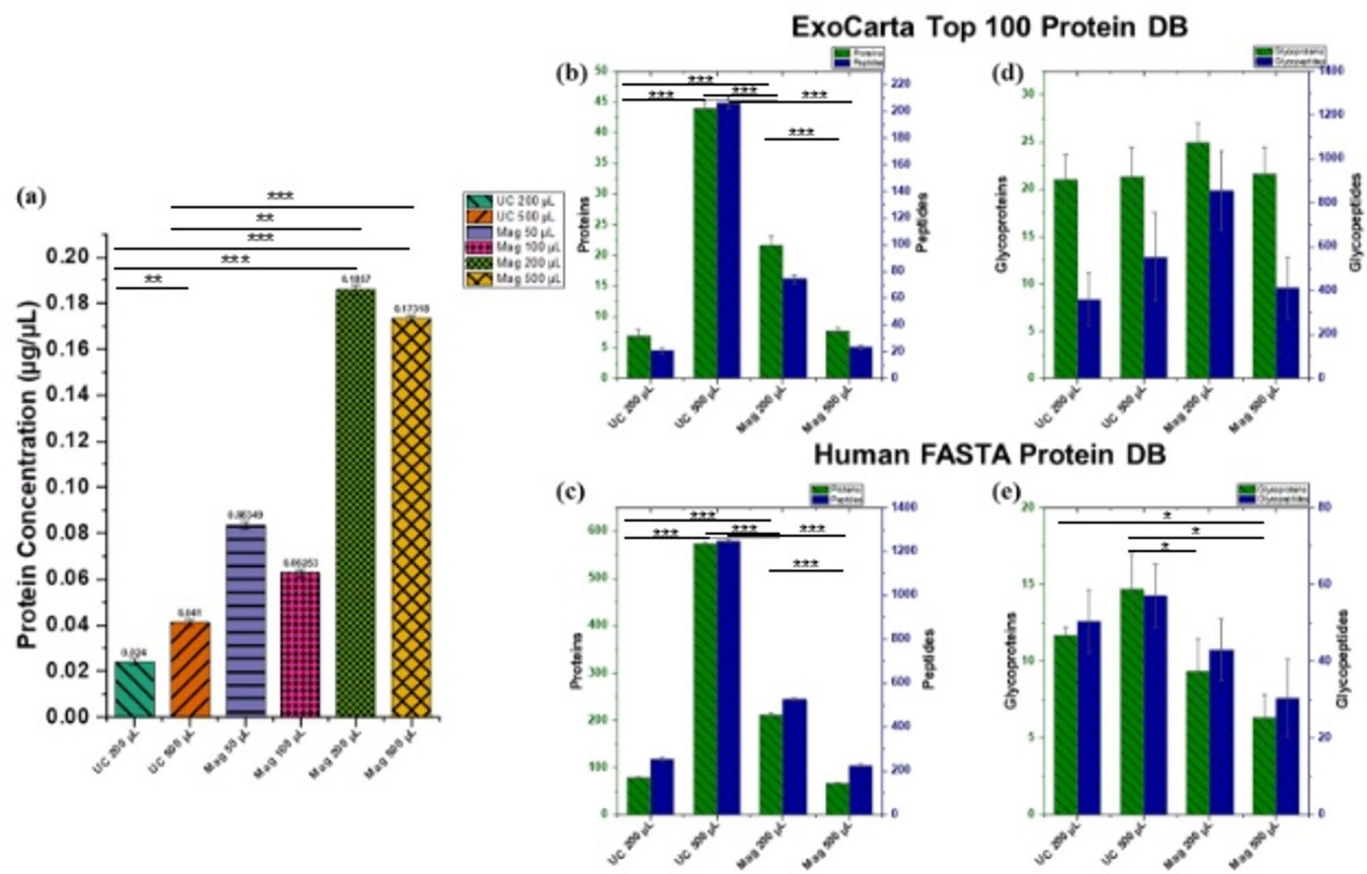
(**a**) Comparison of protein yield across the different sEV isolation methods. Mag-200 produced the highest protein yield, followed by Mag-500, while UC-200 yielded the lowest. Comparison of the number of (**b**) Top 100 EV proteins and peptides, (**c**) total proteins and peptides identified using the UniProt KB/Swiss-Prot human protein database, (**d**) Top 100 EV glycoproteins and glycopeptides, and (**e**) total glycoproteins and glycopeptides identified using the UniProt KB/Swiss-Prot human protein database across the different isolation methods. Asterisks indicate statistically significant differences (*p < 0.05; **p < 0.01; ***p < 0.001). *N* = 3.

We assessed the total protein yield from the isolated sEVs using the Micro-BCA protein assay. As shown in Fig. 5a, the Mag-200 method produced the highest protein yield, followed closely by Mag-500, while both UC methods resulted in relatively lower yields. Additional analysis of smaller sample volumes (50 µL and 100 µL) from Mag revealed lower but measurable protein concentrations. Interestingly, the Mag-50 µL fraction yielded slightly more protein than Mag-100 µL. This non-linear trend likely reflects the increased sensitivity of small-volume workflows to minor technical variation, where even slight inconsistencies in pipetting, bead recovery, or elution can disproportionately impact protein yield. These effects are compounded by the detection sensitivity limits of the Micro-BCA assay at low protein concentrations and are less pronounced at higher input volumes, where protein abundance is more robust. Additionally, the comparable protein yields observed between Mag-200 µL and Mag-500 µL may be attributed to a saturation effect of the magnetic beads used in the isolation kit. At higher input volumes, the fixed quantity of beads may become limiting, resulting in reduced binding efficiency and incomplete capture of sEVs. These factors likely explain why increasing the input volume beyond 200 µL did not result in a proportional increase in protein yield.

We further characterized sEV purity by evaluating marker proteins and contaminants within the proteomic and glycoproteomic profiles. Figure 5b,d display the number of ExoCarta top 100 EV marker proteins/glycoproteins and identified peptides/glycopeptides across different isolation methods. In Fig. 5c,e, the total number of proteins/glycoproteins and peptides/glycopeptides identified using the UniProt KB/Swiss-Prot human protein database are presented.

UC-500 yielded the highest number of EV-related proteins, followed by Mag-200, whereas UC-200 and Mag-500 contained notably fewer EV-related proteins (Fig. 5b). A similar trend was observed when analyzing total protein content using the complete human protein database (Fig. 5c). In contrast, the number of EV-related glycoproteins was comparable across all fractions (Fig. 5d). UC-500 outperformed other methods in terms of total glycoproteins identified using the full human protein database, although this was not statistically different from UC-200 (Fig. 5e). A complete list of identified proteins, glycoproteins, and glycopeptides is provided in Online Resource 5.

**Fig. 6:**
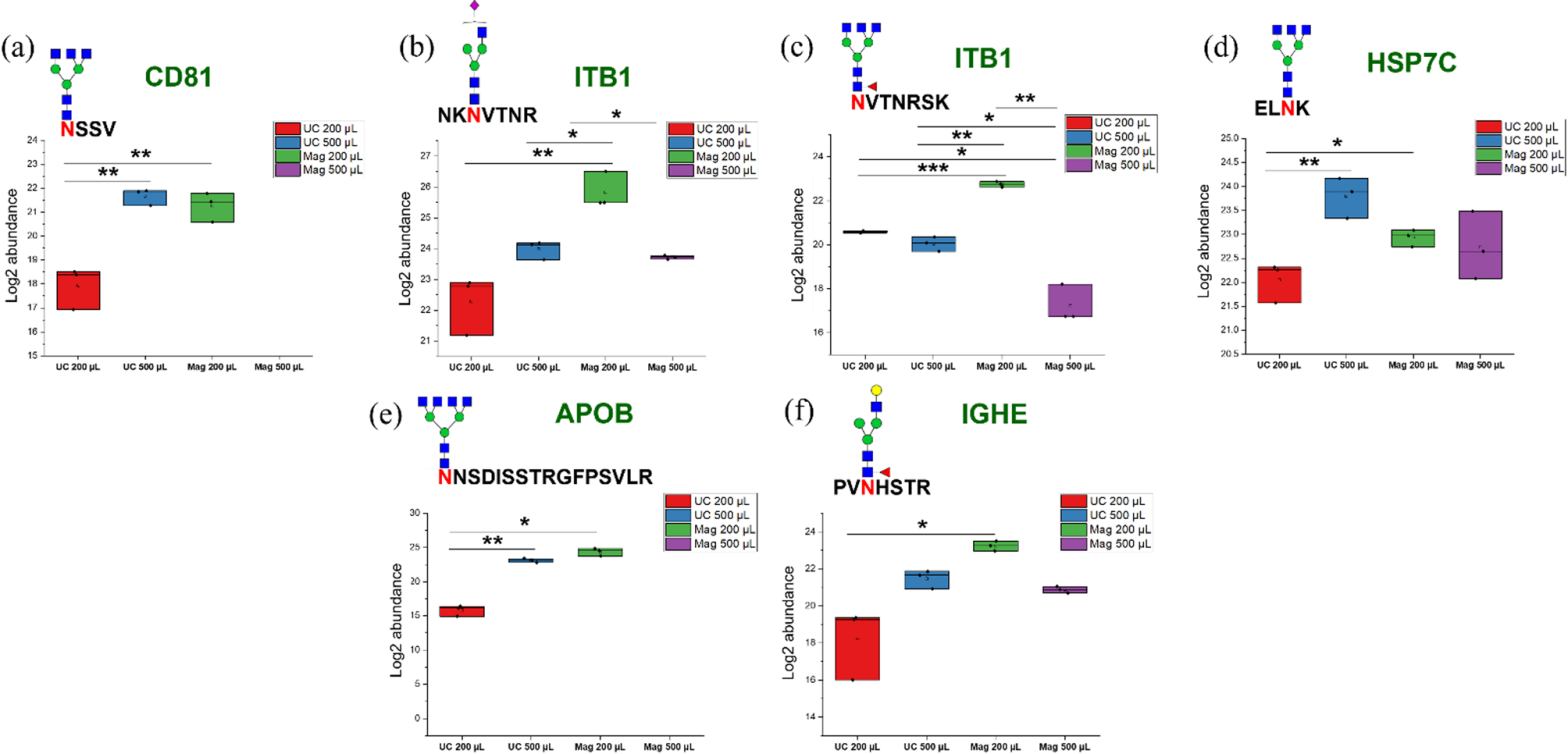
Quantitative distribution of selected glycopeptides derived from (**a**–**d**) sEV marker proteins and (**e**,**f**) serum sEV contaminants across the different isolate fractions. Each isolation method yielded varying levels of glycopeptides from marker proteins CD81, ITB1, and HSP7C, as well as contaminants APOB and IGHE. Glycopeptide abundance was calculated based on peak area and log-transformed to facilitate comparison across methods. Asterisks indicate statistically significant differences (* *p* < 0.05, ** *p* < 0.01, *** *p* < 0.001). Symbols: *blue square*, N-acetylglucosamine (GlcNAc); *yellow circle*, Galactose (Gal); *red inverted triangle*, Fucose (Fuc); *green circle*, Mannose (Man); *blue circle*, Glucose (Glc); *violet diagonal*, N-acetylneuraminic acid (NeuAc/Sialic Acid). *N* = 3.

Additionally, we evaluated glycopeptides associated with specific marker proteins and contaminants in all fractions. Varying levels of glycopeptides from markers CD81, ITB1, and HSP7C (Fig. 6a–d), as well as contaminants such as APOB and IGHE (Fig. 6e,f), were detected in most fractions. Notably, the Mag-500 fraction did not contain quantifiable levels of the CD81 glycopeptide.

#### Cellular component enrichment analysis of sEV proteins

The proteomics dataset, generated from the complete human protein database, was analyzed for cellular component enrichment using the Gene Ontology (GO) database within FunRich 3.1.3 software^62,69^. The proteins identified across the different sEV fractions were predominantly associated with the “extracellular exosome” GO term (GO: 0070062). Among the groups, Mag-500 displayed the highest association, with 67.2% of its total proteins annotated to this term, followed by UC-200 (63.8%), Mag-200 (61.8%), and UC-500 (45.7%).

In addition to exosome-related proteins, many mapped proteins showed enrichment in other cellular components, such as the cytosol, nucleus, and plasma membrane. Notably, 0.2% of proteins from the UC-500 fraction were associated with the "low-density lipoprotein particle" GO term (GO:0034362). A detailed breakdown of the top six enriched GO cellular component terms, along with the percentages of lipoprotein association, is provided in Online Resource 6, Table 1.

#### Impact of isolation method and sample volume on glycoprotein and glycopeptide identification

The isolation method used for sEVs influences their protein, DNA, and RNA composition^70^. We compared glycoproteomic datasets using Venn diagrams to investigate the variations in glycoproteins and glycopeptides across different isolation techniques and sample volumes.

Our analysis revealed that eleven ExoCarta glycoproteins were shared between the Mag-200 and Mag-500 methods, constituting 30% and 28% of their respective glycoproteomes (Online Resource 6, Fig. 1a). In contrast, UC-200 and UC-500 shared 26 glycoproteins, representing 74% and 70% of their glycoproteomes, respectively (Online Resource 6, Fig. 1b). Notably, 86% of the UC-200 glycoproteome overlapped with Mag-200, whereas Mag-200 shared 63% of its glycoproteome with UC-200 (Online Resource 6, Fig. 1c). At higher starting sample volumes, UC-500 and Mag-500 displayed considerable similarity, sharing 77% and 72% of their glycoproteins, respectively (Online Resource 6, Fig. 1d).

We also observed marked differences in glycopeptide profiles across the various isolation methods and sample volumes (Online Resource 6, Fig. 2a–d). When analyzing the complete human protein database, distinct glycoproteomes emerged among the isolation methods, with common glycoproteins ranging from 11 to 54% (Online Resource 6, Fig. 3a–d). Additionally, substantial variability was found in the glycopeptides identified across the different experiments (Online Resource 6, Fig. 4a–d).

Notably, the number of glycopeptides identified using the ExoCarta-based search (Online Resource 6, Fig. 2) was higher than those identified using the full UniProt/Swiss-Prot human proteome database (Online Resource 6, Fig. 4). This discrepancy is primarily due to the smaller search space in the curated ExoCarta database, which enhances the statistical confidence of glycopeptide-spectrum matches and enables more identifications to pass the 1% false discovery rate (FDR) threshold. In contrast, the broader UniProt/Swiss-Prot human proteome database increases the potential for ambiguous matches and false positives, resulting in more stringent FDR filtering and ultimately fewer glycopeptide identifications.

To substantiate the validity of our glycopeptide identifications, we have included a representative MS2 spectrum in the supplementary materials (Online Resource 6, Fig. 5), depicting a glycopeptide derived from Haptoglobin in the UC-500 fraction. This spectrum clearly displays diagnostic fragment ions from both the glycan and peptide backbone, reinforcing the robustness of our glycoproteomic assignments.

#### Site-specific glycan microheterogeneity in serum sEV glycoproteins

Glycosylation, a complex post-translational modification (PTM), can occur at multiple sites on a protein, leading to a wide range of glycan structures. Site-specific glycan microheterogeneity refers to the variation in glycan types and structures at individual glycosylation sites, which can influence biological processes such as protein–protein interactions, immune recognition, and cellular signaling. Understanding this microheterogeneity is critical for uncovering its impact on protein function and its potential implications for health and disease.

**Fig. 7:**
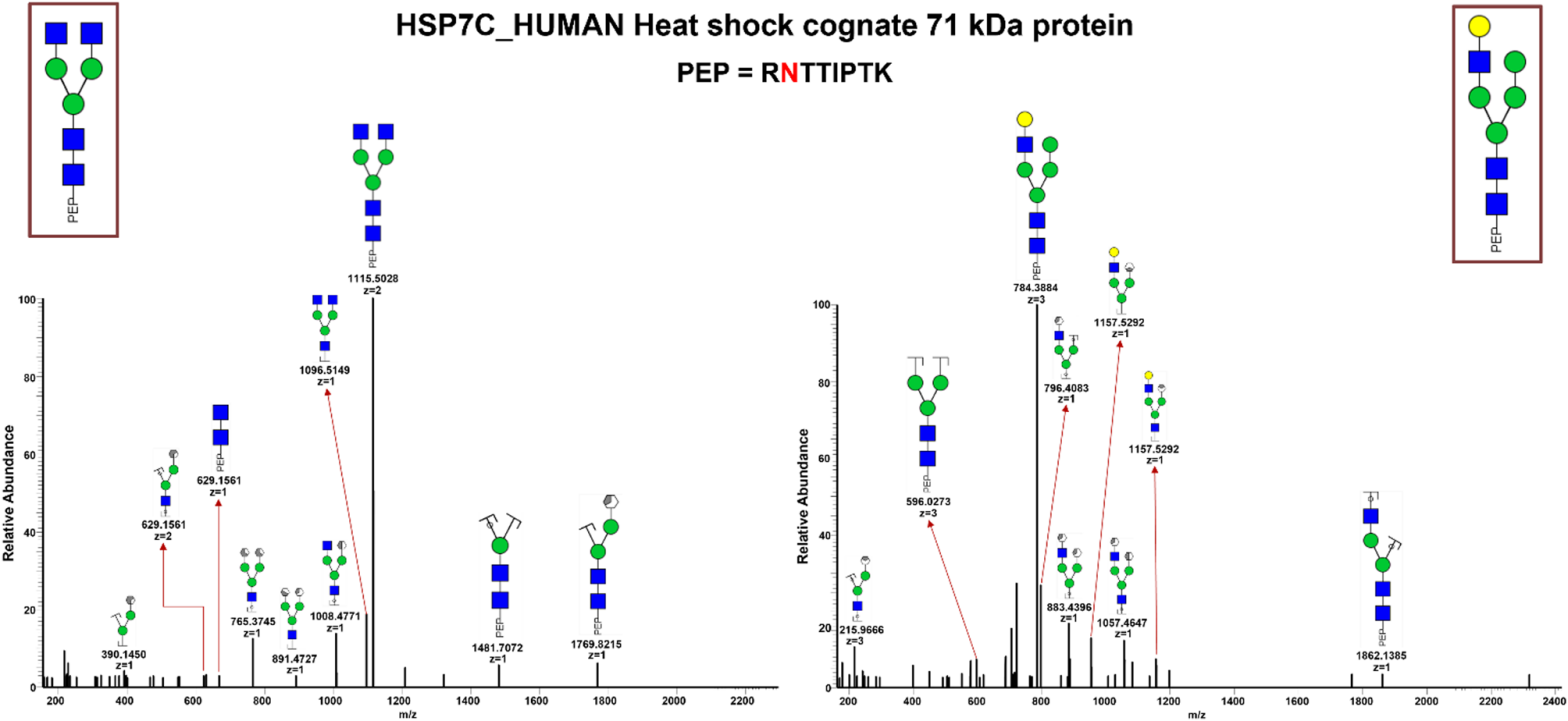
HCD tandem mass spectra of two tryptic glycopeptides with *N*-glycans—(**a**) HexNAc_4_Hex_3_ and (**b**) HexNAc_3_Hex_5_ – from marker protein, HSP7C, illustrating site-specific *N*-glycan microheterogeneity in serum sEV glycoproteins. Ion intensities are normalized to the base peak of each individual MS/MS spectrum. Symbols and glycan color codes as in Fig. 6

This study investigated site-specific glycan microheterogeneity in serum sEV glycoproteins, using the human heat shock cognate 71 kDa protein (HSP7C) as a model. We identified two distinct glycan structures, HexNAc4Hex3 and HexNAc3Hex5, attached to the Asn-417 residue of HSP7C. Figure 7a,b display the MS/MS spectra of the glycoforms, illustrating the site-specific microheterogeneity observed in our analysis.

## Discussion

This study provides a comprehensive glycoproteomic comparison of serum-derived sEVs isolated using differential ultracentrifugation (UC) and the MagCapture Exosome Isolation Kit PS Ver. 2 (Mag) across two starting volumes (200 µL and 500 µL). While previous studies have compared sEV isolation methods based on protein or RNA content^35–39^, our work uniquely focuses on how isolation strategy and input volume influence the glycoproteomic yield and quality of serum sEVs. Through systematic comparisons, we reveal notable differences in sEV yield, size distribution, protein recovery, and glycoproteomic profiles depending on the isolation method and input volume. These findings advance the current understanding of methodological impacts on sEV cargo composition, offering practical insights for optimizing sEV isolation in biomarker discovery and therapeutic applications. Our analytical workflow, adhering to MISEV2018 guidelines^65^, integrated high-resolution TEM imaging, nano-flow cytometry (nFCM), LC–MS/MS proteomic analysis, and detailed *N*-glycoproteomic profiling.

Ultracentrifugation remains the most common sEV isolation technique, though it has several limitations. It is time-consuming, requires specialized equipment, and is less effective for extracting sEVs from small sample volumes or viscous biofluids^55^. To address this, we diluted the serum samples with an equal volume of PBS before extraction^71^. TEM images from both UC and Mag methods confirmed the presence of intact sEVs with typical cup-shaped morphology and sizes ranging from 40 to 200 nm, consistent with previous reports^66–68^.

Our results show that UC-500 produced larger sEVs compared to Mag-200, which yielded the highest particle concentration. These findings align with previous reports from Helwa et al.^72^, who observed that UC-extracted sEVs from pooled serum exhibited larger particles than those obtained from commercial kits. This size difference may be attributed to vesicle aggregation during ultracentrifugation^73^. Furthermore, nFCM analysis showed that particle concentration and size distribution varied with both isolation method and starting sample volume, with UC-500 and Mag-500 producing larger particles than their respective 200 µL counterparts. This observation suggests that processing larger input volumes may favor the enrichment of a broader or more heterogeneous sEV population, potentially including a greater proportion of larger vesicles or aggregates.

Although Mag-200 produced the highest particle yield and protein concentration (Figs. 4a and 5a), subsequent proteomic analysis indicated that it did not achieve the highest sEV purity. This is evidenced by the lower number of EV marker proteins identified from the ExoCarta database search (Fig. 5b), and the elevated levels of contaminating glycopeptides derived from APOB and IGHE (Fig. 6e,f), compared to UC-500. The presence of these high-abundance serum contaminants may interfere with downstream glycoproteomic analysis, reduce the detectability of low-abundance sEV proteins, and ultimately compromise the specificity of potential biomarker discovery efforts. Moreover, the immunoaffinity method of the MagCapture kit leverages the high affinity of T-cell immunoglobulin domain and mucin domain-containing protein 4 (Tim4) protein for phosphatidylserine, which is broadly present on the surface of EVs. Consequently, the Tim4-affinity method is unable to differentiate between smaller and larger EVs present in the sample^34^. This increases the likelihood for the capture of larger vesicles that were not completely excluded during the sample pre-treatment step, resulting in a completely heterogeneous population of sEVs.

In a similar study using conditioned cell culture media, Patel et al.^74^ reported high expression of the microvesicle marker ARF-6 in sEV preparations isolated with various commercial kits, including MagCapture. They found that UC-based sEVs isolation protocol had the highest purity and least contamination from larger microvesicles. Correspondingly, the higher number of EVs-related proteins we observed for UC-500 suggests better enrichment of sEVs and separation from larger EVs compared to the Mag methods.

In contrast to our findings for UC-200, Brennan et al.^75^ reported very high protein content and particle numbers which could not be definitively linked to sEVs through robust proteomic characterization, as performed in this study. The efficiency of UC-sEVs isolation is often challenged by the frequent co-isolation of protein aggregates and non-sEVs particles like lipoproteins of similar sizes and densities, thus resulting in a possible overestimation of protein content^76^. This issue is even more apparent when limited sample volumes are used with UC, resulting in reduced sEV yields and increased background contamination.

Gene ontology (cellular component) analysis in our study confirmed a strong association of the identified proteins with the “extracellular exosome” GO term, validating the enrichment of sEVs to varying extents. While UC-500 had the lowest enrichment score for the extracellular exosome GO term, it produced more protein IDs compared to other methods. As sEV research progresses, the proteome attributed to vesicles continues to expand, suggesting that our dataset may reveal novel sEV-associated proteins. Interestingly, the detection of low-density lipoprotein (LDL) particle-associated proteins in UC-500 fractions points to co-isolation either as contaminants or as components of an emerging structure known as the EV 'corona’, a phenomenon documented in numerous studies^35,70,76–81^. Since the release of the MISEV2018 guidelines^65^, there has been growing recognition that specific molecules and naturally occurring non-EV particles, such as lipoproteins, can adhere to the surface of EVs, forming EVs coronas that perform specific functions^82–84^.

Glycoproteomic analysis revealed differences in glycoprotein and glycopeptide profiles based on the isolation technique and starting sample volume. Venn diagram comparisons showed varying degrees of overlap across methods and volumes, highlighting methodological influences on the composition of the isolated sEV cargo. UC-200 and Mag-200 shared a substantial proportion of their glycoproteomes (Online Resource 6, Fig. 1c), and a comparable level of overlap was observed between UC-500 and Mag-500 (Online Resource 6, Fig. 1d), suggesting that both low- and high-volume inputs can yield consistent glycoprotein profiles across the isolation strategies in the context of EV-specific glycoproteins. UC-500 achieved the highest number of glycoprotein identifications in the full human protein database search, although this was not statistically different from UC-200 (Fig. 5e). In contrast, Mag-200 yielded the highest number of glycoprotein identifications in the smaller, ExoCarta-based search, based on observational trends (Fig. 5d). Notably, Mag-200 also exhibited a significantly higher number of EV-related proteins than UC-200 (Fig. 5b), suggesting better enrichment for vesicle-specific cargo and supporting its viability as an alternative to UC-500 for comprehensive glycoproteomic profiling when sample volume is limited. Although UC-500 had a significantly lower protein yield than Mag-200 (Fig. 5a), it produced more protein and peptide identifications from the larger database search, reinforcing its suitability for proteomic studies. Peptides are more prevalent and chemically less complex than glycopeptides, resulting in higher ionization efficiency and improved detectability by mass spectrometry, which facilitates easier identification and quantification even at low concentrations. Moreover, the likely co-isolation of microvesicles and elevated levels of contaminating glycopeptides in Mag-200 preparations (Fig. 6) may contribute to the lower numbers of protein and peptide identifications relative to UC-500.

EV glycoproteomic studies face several challenges, including the complexity of glycosylation, the low abundance of sEVs in biofluids, and contamination with abundant serum proteins. Glycopeptide enrichment techniques are often necessary but can lead to sample loss and further reduce detectable quantities. Given these challenges, fewer studies have focused on sEV glycoproteomics. The workflow presented in this study can guide the selection and refinement of isolation techniques for proteomic and glycoproteomic applications, particularly in biomarker development.

Finally, our analysis of site-specific glycan microheterogeneity, using HSP7C as an example, revealed distinct glycoforms, highlighting the biological significance of glycosylation patterns in serum sEV glycoproteins. Understanding these patterns is essential for exploring the role of sEV glycans in intercellular communication and disease processes.

In summary, this study underscores the substantial influence of isolation techniques and starting sample volume on the yield, purity, and (glyco)proteomic composition of sEVs. Differential ultracentrifugation, particularly with larger sample volumes (500 µL), offered better and more consistent enrichment of sEV proteins and glycopeptides despite some contamination risks. In contrast, the MagCapture immunoaffinity kit yielded higher particle numbers and is a practical option for *N*-glycoproteomic analysis when sample availability is limited. However, its tendency to co-isolate larger vesicles highlights the need for further refinement to achieve higher purity.

In future studies, the integration of immunodepletion strategies targeting high-abundance serum proteins such as albumin and immunoglobulins, as well as lipoprotein particles, could further enhance the depth and sensitivity of both sEV proteomic and glycoproteomic analyses. These abundant components often co-isolate with sEVs and dominate the peptide pool during LC–MS/MS, thereby limiting the detection of low-abundance, biologically relevant sEV-specific proteins and glycopeptides. This limitation is particularly critical in glycoproteomics, where glycopeptides are inherently low in abundance and exhibit considerable microheterogeneity, making them especially prone to ion suppression. A recent study demonstrated that immunodepletion of plasma prior to sEV isolation by size exclusion chromatography improved proteome coverage, identifying more proteins in EVs from depleted plasma versus non-depleted plasma, while also increasing the particle-to-protein ratio^85^. Notably, this improvement was achieved using only 100 µL of plasma, emphasizing the clinical feasibility of the approach. However, the study also reported a slight reduction in the detection of canonical EV markers in the depleted group, indicating a trade-off between proteome depth and specificity. Incorporating similar depletion methods into our workflow could improve analytical resolution and enable more comprehensive profiling of the sEV cargo, though future work is needed to optimize conditions that balance enhanced depth with retention of EV-defining features.

Overall, this study provides an adaptable analytical workflow and practical recommendations for optimizing sEV isolation protocols based on specific omics applications. These findings improve the reliability and reproducibility of sEV-based research and hold particular promise for advancing biomarker discovery and therapeutic development.

## Electronic supplementary material

Below is the link to the electronic supplementary material.

Supplementary Material 1

## Data Availability

The mass spectrometry proteomics and glycoproteomics data have been deposited in the MassIVE partner repository under project ID MSV000095807.
