# Supplementary material for: Glycoproteomic profiling of serum-derived small extracellular vesicles enriched via ultracentrifugation and affinity-based techniques
Source: Sci Rep. 2025 Jul 1;15:21565. doi: 10.1038/s41598-025-05430-1 (PMC12218995; doi:10.1038/s41598-025-05430-1)
Supplement: Supplementary file 1 — Supplementary Material 1 [file 41598_2025_5430_MOESM1_ESM.zip › ESM_1.pdf]

Size & Concentration Report

UC 200

Data File	20240201 UC 200 3.nfa	Population	Total

SN: FNAU30T22111554
Software: V2.0
Sample Pressure: 1.0Kpa

Laser: 10/50 mW 488
SS Decay: 10%
Threshold/sub: 72.6 10.1 1.8 1/0 0 0 0
Min Width: 0.3 ms

Total Size Information

All Events	52	
Gating Events	52	
% of all	100.00	
Median	52.2	nm
Mean	71.6	nm
Std Dev.	48.3	nm

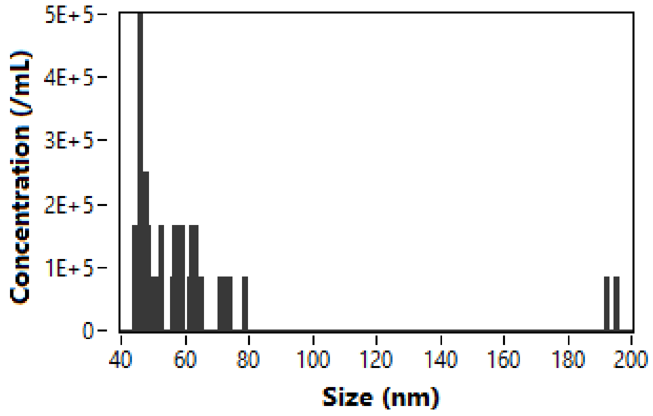

Total Concentration Information

	Particle Number	Dilution Factor
STD	2610	100
Blank	104	———
Sample	156	1

STD Con.	2.17E+10	Particles/mL
Sample Flow Rate	12.03	nL/min
Sample Con.	4.32E+6	Particles/mL

Corrected Ratio: 52/52 100.0%

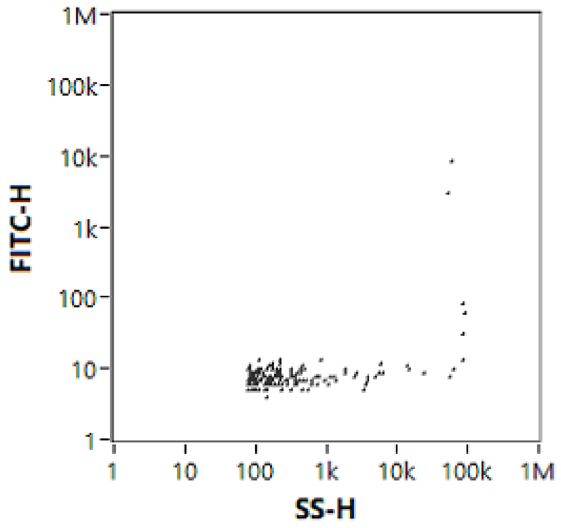

Report By :

2/1/2024 6:30 PM
