# Supplementary material for: Glycoproteomic profiling of serum-derived small extracellular vesicles enriched via ultracentrifugation and affinity-based techniques
Source: Sci Rep. 2025 Jul 1;15:21565. doi: 10.1038/s41598-025-05430-1 (PMC12218995; doi:10.1038/s41598-025-05430-1)
Supplement: Supplementary file 1 — Supplementary Material 1 [file 41598_2025_5430_MOESM1_ESM.zip › ESM_4.pdf]

Size & Concentration Report

Mag 500

Data File	20240201 Mag 500 6.nfa	Population	Total

SN: FNAU30T22111554
Software: V2.0
Sample Pressure: 1.0Kpa

Laser: 10/50 mW 488
SS Decay: 10%
Threshold/sub: 72.6 10.1 1.8 1/0 0 0 0
Min Width: 0.3 ms

Total Size Information

All Events	225	
Gating Events	225	
% of all	100.00	
Median	77.2	nm
Mean	90.6	nm
Std Dev.	37.8	nm

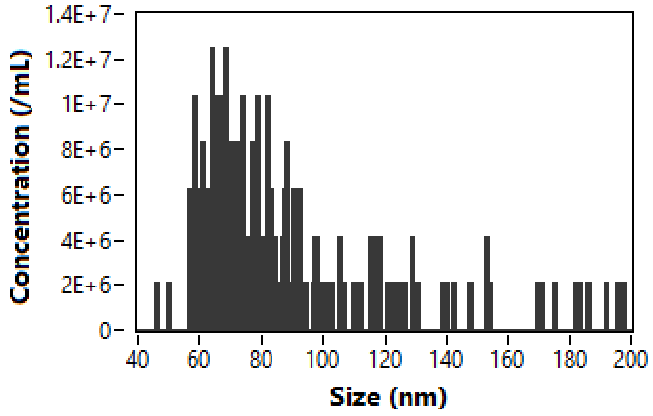

Total Concentration Information

	Particle Number	Dilution Factor
STD	2610	100
Blank	104	——
Sample	329	25

STD Con.	2.17E+10	Particles/mL
Sample Flow Rate	12.03	nL/min
Sample Con.	4.68E+8	Particles/mL

Corrected Ratio: 225/225 100.0%

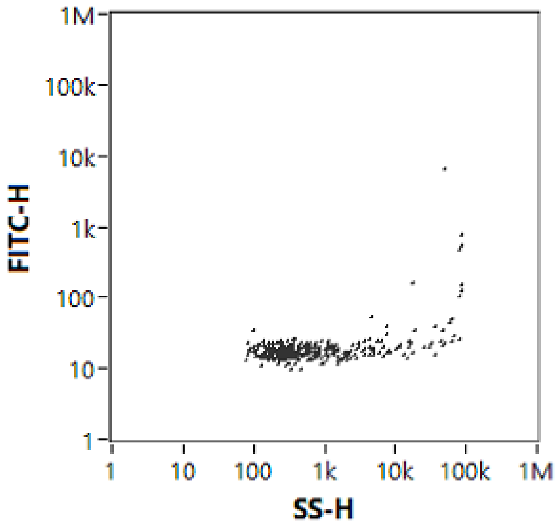

Report By :

2/1/2024 6:33 PM
