# Supplementary material for: Glycoproteomic profiling of serum-derived small extracellular vesicles enriched via ultracentrifugation and affinity-based techniques
Source: Sci Rep. 2025 Jul 1;15:21565. doi: 10.1038/s41598-025-05430-1 (PMC12218995; doi:10.1038/s41598-025-05430-1)
Supplement: Supplementary file 1 — Supplementary Material 1 [file 41598_2025_5430_MOESM1_ESM.zip › ESM_6.pdf]

[Supplementary Information]

Glycoproteomic Profiling of Serum-derived Small Extracellular Vesicles Enriched via Ultracentrifugation and Affinity-Based Techniques

Mojibola Fowowe [1], Cristian D Gutierrez Reyes [1], Judith Nwaiwu [1], Joy Solomon [1], Oluwatosin Daramola [1], Sherifdeen Onigbinde [1], Joseph Andrew Whitley [2], Houjian Cai [2], and Yehia Mechref [1]*

[1] Department of Chemistry and Biochemistry, Texas Tech University, Lubbock, TX 79409-1061

mfowowe@ttu.edu (M.F.); cristian.d.gutierrez-reyes@ttu.edu (C.D.G); jnwaiwu@ttu.edu (J.N.); joy.solomon@ttu.edu (J.S.); odaramol@ttu.edu (O.D.); sonigbin@ttu.edu (S.O.); yehia.mechref@ttu.edu (Y.M.)

[2] Department of Pharmaceutical & Biomedical Sciences, College of Pharmacy, University of Georgia, Athens, GA 30602

Joseph.Whitley@uga.edu (J.A.W.); caihj@uga.edu (H.C.)

***Corresponding Author**

Department of Chemistry and Biochemistry

Texas Tech University

Lubbock, TX 79409-1061

Email: Yehia.Mechref@ttu.edu

Tel: 806-742-3059

Fax: 806-742-1289

Keywords: Small extracellular vesicle, EVs, immunoaffinity capture, ultracentrifugation, glycoproteomics, proteomics

Table of Contents:

Supplementary Figures:

Fig. 1 Comparison and overlap of the ExoCarta top 100 EVs glycoproteins identified from the isolation methods

Fig. 2 Comparison and overlap of the ExoCarta top 100 EVs glycopeptides identified from the isolation methods

Fig. 3 Comparison and overlap of glycoproteins identified from the UniProt KB/Swiss-prot human protein database among the different isolation methods

Fig. 4 Comparison and overlap of glycopeptides identified from the UniProt KB/Swiss-prot human protein database among the different isolation methods

Fig. 5. Representative MS2 spectrum of a glycopeptide derived from Haptoglobin, isolated from the UC-500 fraction. The spectrum displays diagnostic fragment ions from both the peptide backbone and glycan moiety, supporting confident glycopeptide identification

Supplementary Tables:

Table 1 Functional enrichment analysis (cellular component) of the top six gene ontology (GO) terms and percentage of lipoprotein association within the dataset

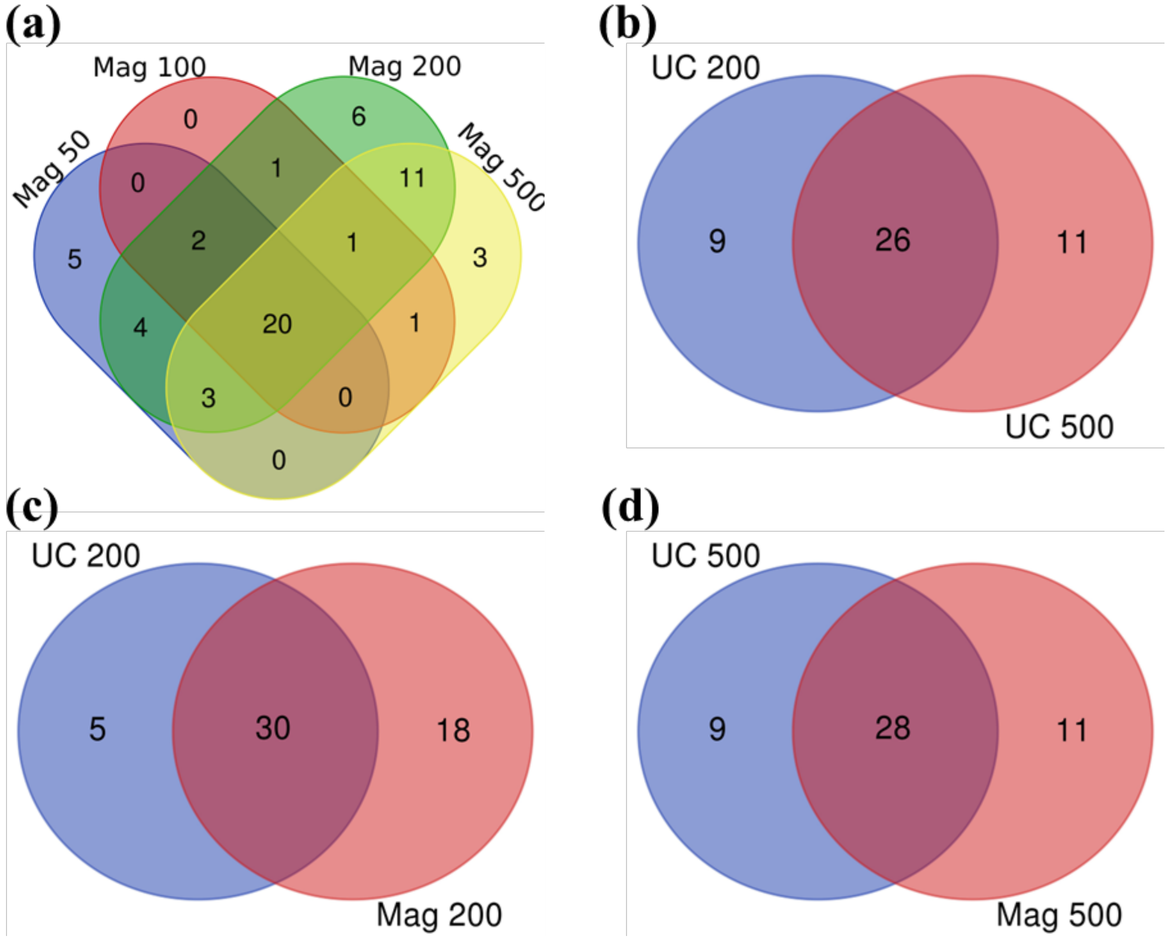

Fig. 1 Comparison and overlap of the ExoCarta top 100 EVs glycoproteins identified from the isolation methods. Results are based on a combined analysis of all replicates for each method

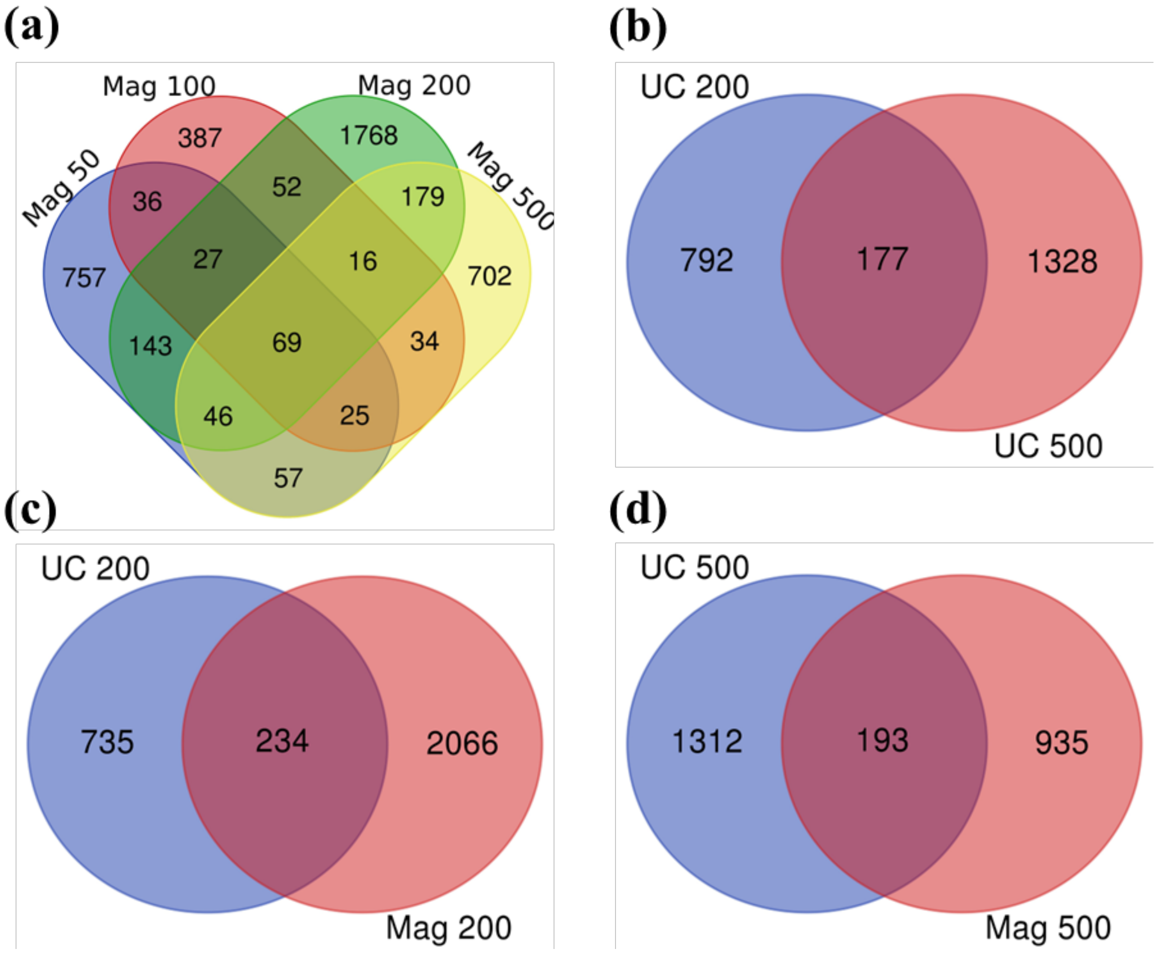

Fig. 2 Comparison and overlap of the ExoCarta top 100 EVs glycopeptides identified from the isolation methods. Results are based on a combined analysis of all replicates for each method

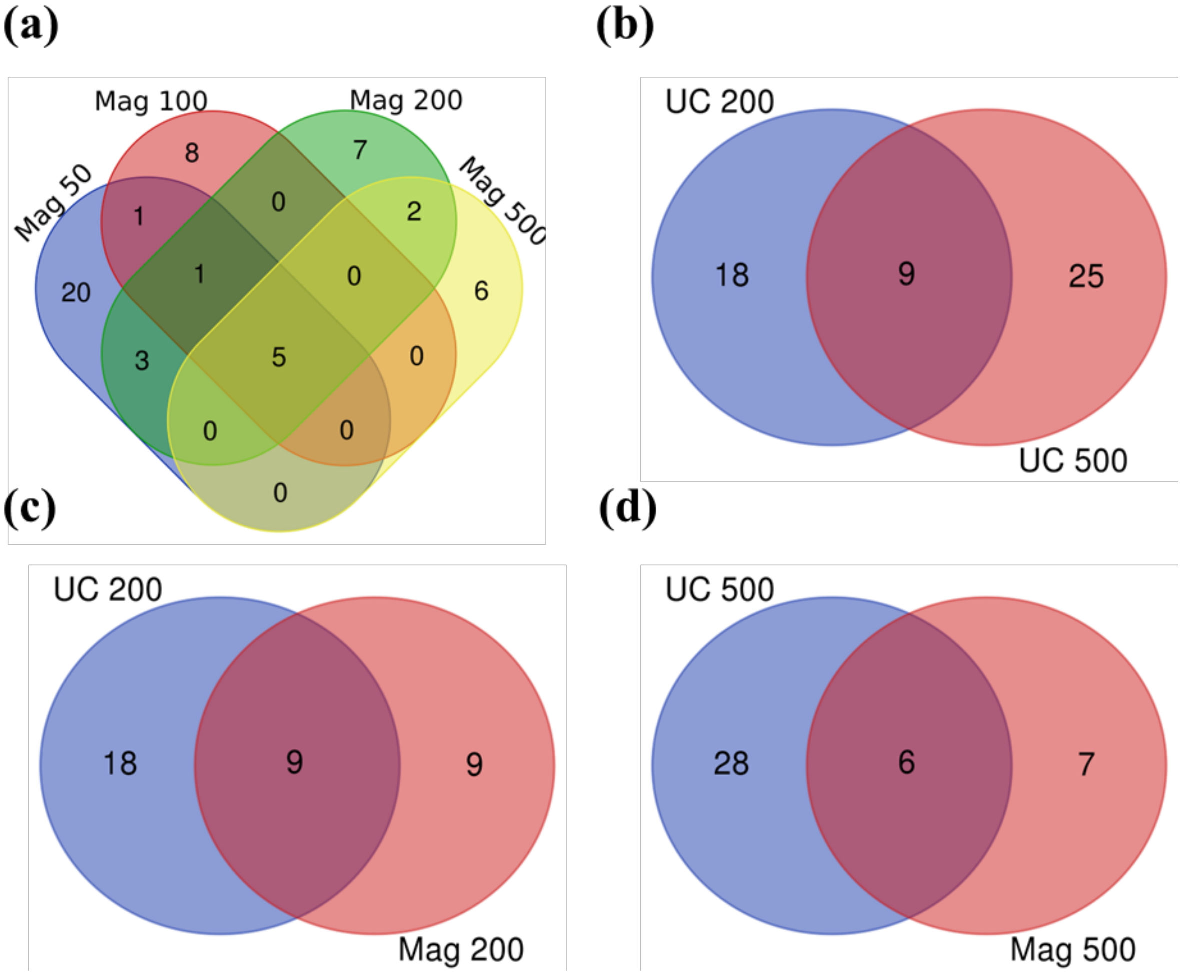

Fig. 3 Comparison and overlap of glycoproteins identified from the UniProt KB/Swiss-prot human protein database among the different isolation methods. Results are based on a combined analysis of all replicates for each method

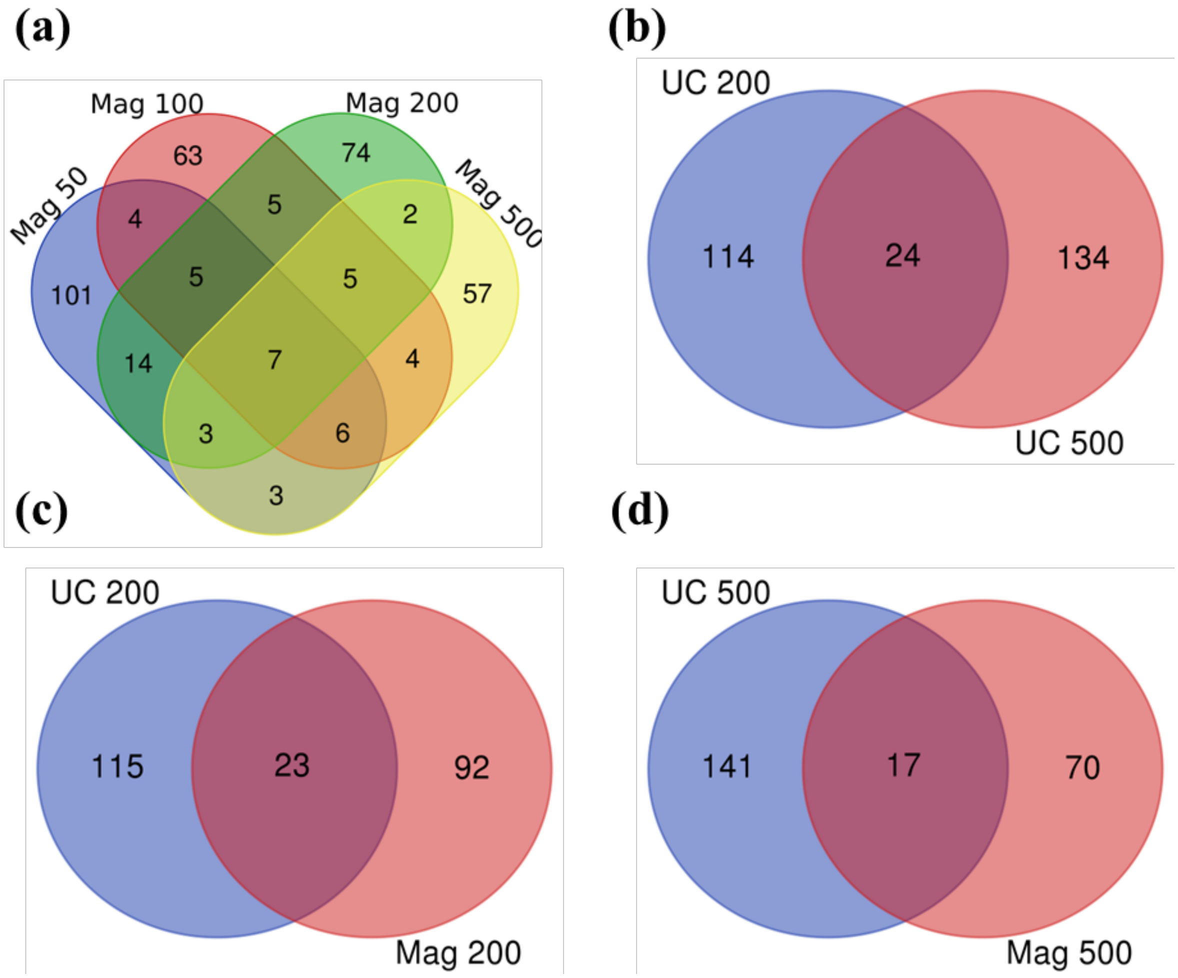

Fig. 4 Comparison and overlap of glycopeptides identified from the UniProt KB/Swiss-prot human protein database among the different isolation methods. Results are based on a combined analysis of all replicates for each method

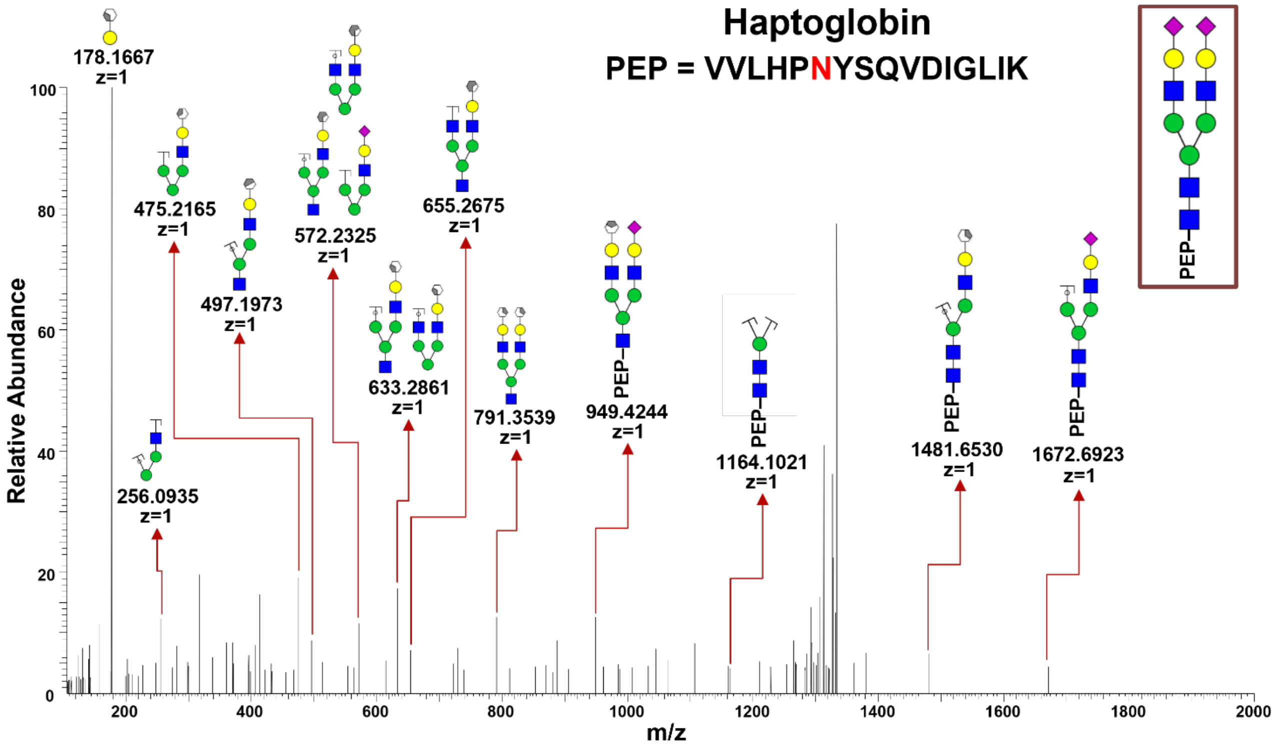

Fig. 5. Representative MS2 spectrum of a glycopeptide derived from Haptoglobin, isolated from the UC-500 fraction. The spectrum displays diagnostic fragment ions from both the peptide backbone and glycan moiety, supporting confident glycopeptide identification

Table 1. Functional enrichment analysis (cellular component) of the top six enriched GO terms and percentage of lipoprotein association within the dataset

Gene Ontology (Cellular Component)							
Method	% Extracellular vesicular exosome	% Cytosol	% Nucleus	% Cytoplasm	% Plasma membrane	% Membrane	% Low-density lipoprotein particle
UC-200	63.8	61.3	60.0	52.5	37.5	40.0	0.0
UC-500	45.7	50.4	31.7	40.1	34.5	41.2	0.2
Mag-50	-	-	-	-	-	-	-
Mag-100	-	-	-	-	-	-	-
Mag-200	61.8	55.1	39.6	43.0	39.6	42.5	0.0
Mag-500	67.2	59.4	60.9	59.4	45.3	43.8	0.0
